# Conservation of the three-dimensional structure in non-homologous or unrelated proteins

**DOI:** 10.1186/1479-7364-6-10

**Published:** 2012-08-02

**Authors:** Konstantinos Sousounis, Carl E Haney, Jin Cao, Bharath Sunchu, Panagiotis A Tsonis

**Affiliations:** 1 Department of Biology, University of Dayton, Dayton, OH, 45469-2320, USA; 2 Department of Chemical Engineering, University of Dayton, Dayton, OH, 45469-2320, USA; 3 Department of Chemistry, University of Dayton, Dayton, OH, 45469-2320, USA

**Keywords:** 3D protein structure, Conserved motifs, Unrelated proteins

## Abstract

In this review, we examine examples of conservation of protein structural motifs in unrelated or non-homologous proteins. For this, we have selected three DNA-binding motifs: the histone fold, the helix-turn-helix motif, and the zinc finger, as well as the globin-like fold. We show that indeed similar structures exist in unrelated proteins, strengthening the concept that three-dimensional conservation might be more important than the primary amino acid sequence.

## Introduction

When the human genome was sequenced (as well as that of other mammals), it was estimated that there are approximately 25,000 genes encoding for proteins [1,2]. After being synthesized, proteins assume their three-dimensional structure by a specific arrangement of beta strands, alpha helices, turns, or loops. In many cases, a combination of these structural features creates certain motifs, exerting a particular function (i.e., DNA binding) that is quite conserved in proteins from virtually all organisms. Interestingly, the number of these motifs is much smaller than the number of genes. However, it has also been noted that some structural motifs show significant robustness even though no significant homology exists among them at the primary amino acid sequence. It seems that evolutionary constraints have limited the ability of proteins to become vastly different. Moreover, it has been shown that protein structures are three to ten times more conserved than the amino acid sequence [3]. Thus, a particular motif, i.e., a zinc-binding domain of very similar or virtually identical structure, can be found in many different proteins, which could also be unrelated to each other when function is concerned. Thus, it seems that evolution does favor conservation of structural motifs in proteins.

The purpose of this tutorial/review is to illustrate this diversity that exists in the function of structurally conserved protein motifs. For this reason, protein folds with low homology in amino acid sequence and high structural similarity were used. The analysis for the obvious reason of space is not exhaustive and is focused on four specific protein structural folds: We have selected to present data with three different DNA-binding domains: the histone fold, the helix-turn-helix motif (HTH), and the zinc finger, as well the globin-like fold, part of an important protein in oxygen binding and transport. These four folds were chosen because they are ubiquitous in many different organisms and are well represented in many different proteins.

For our comparisons, an intensive search of the Vector Alignment Search Tool (VAST) [4,5], an algorithm to determine three-dimensional (3D) structure similarities according to geometric criteria, was done. A protein family was identified using a representative protein and, using VAST and the Molecular Modeling Database [6], dissimilar structure proteins were identified and annotated followed by root mean square deviation (RMSD) determination. The structures were then downloaded into Cn3D (‘see in 3D’) [7] for viewing the sequence alignment. The above are part of Entrez [8,9]. These structures were then aligned in PyMOL [10] for 3D viewing. The files for the PyMOL structures provided have been downloaded from the Protein Data Bank (PDB) [11]. The lower the RMSD means better structural alignment. Lower identity means that the two proteins do not share the same amino acids in the corresponding structural alignment. Though, depending on how big are the structures that we are comparing, the RMSD and sequence identity may vary. Small domains may contain always certain amino acids increasing the identity. On the other hand, big proteins may not align well and may increase the RMSD. For the present analysis, we chose to set the limits as follows: RMSD to be lower than 3.5 and amino acid identity to be lower than 25% in order to conclude that this pair of proteins has similar structures but dissimilar sequences.

### Globin-like fold

**Figure 1:**
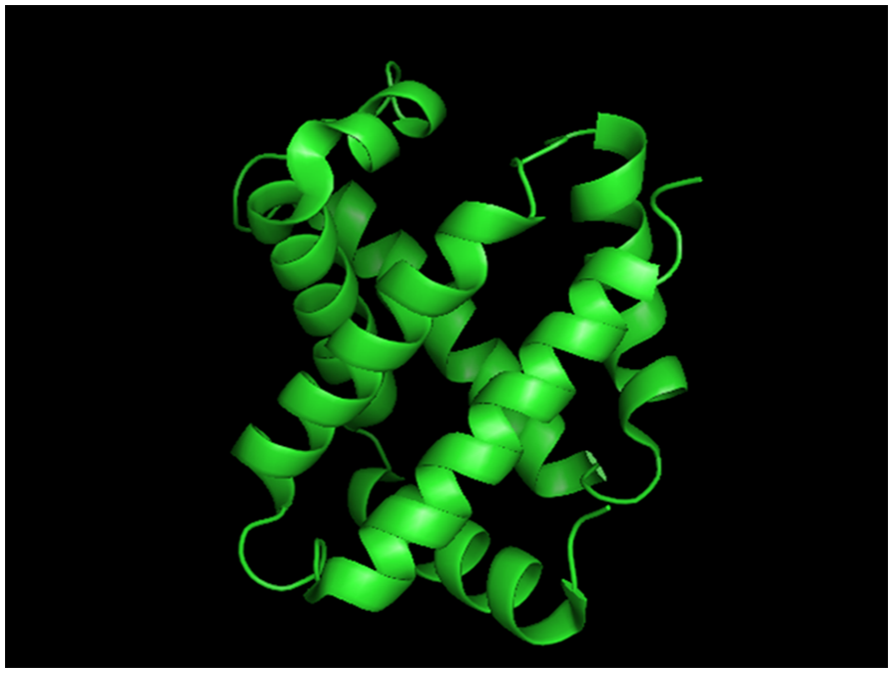
**Human hemoglobin (PDB: 2DN2; chain A) **[13].

Globin-like fold is an all-alpha protein fold normally consisting of six alpha helices [12]. The number of helices can be altered in different families of globin-like proteins. These helices are not randomly distributed in the protein, but they are oriented following standard helix-helix packing rules in order to form a globular structure. Globin-like fold is mostly known from hemoglobins (Figure 1) and myoglobins which play an important role in transferring oxygen to all the tissues of an organism with the help of heme groups which can bind oxygen reversibly. The heme-binding proteins are part of the actual family of globins [12].

The globin family was the first example that showed structural conservation even in different organisms [14-17] and led scientists' pursuit to prove that 3D structures of proteins are more conserved than their sequences. It turned out that globin-like folds exist in many proteins with different functions. Hemoglobins and myoglobins play a role in oxygen transport; cyanoglobins [18] bind to oxygen to help in cellular processes; phycocyanins and phycoerythrins [19,20] play a role in absorbing light; cytokines and immuno-globins [21,22] play a role in the immune system; and fibronectin [23] is part of the extracellular matrix. Natural selection kept the 3D structure of this fold intact [24] while utilizing it for different functions to meet other required organismal requirements.

**Figure 2:**
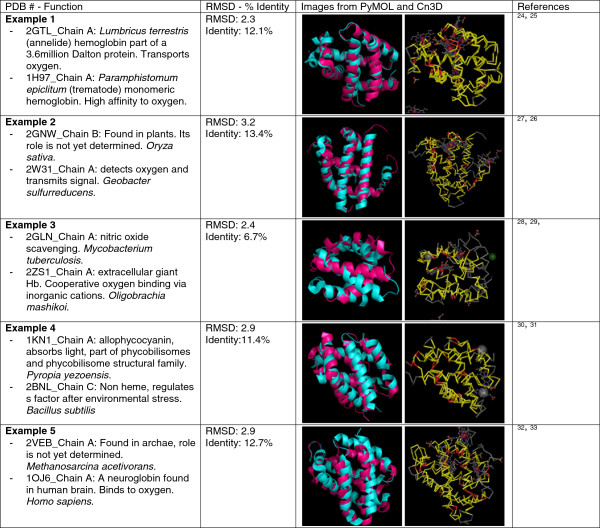
**Comparison of structure and sequence similarity of sample globin-like fold proteins according to PDB number.** First column: PDB number and a brief description of the protein. Second column: RMSD and amino acid sequence identity as defined by VAST. Third column: Left is the alignment of the two proteins taken by PyMOL. In the structure representation, the first protein is in *pink*, and the second, in *cyan*. Right is the alignment of the two proteins taken by Cn3D. In the sequence representation, *red* indicates the same amino acid, whereas *yellow* indicates differing amino acids. Fourth column: references.

We have compared pairs of functionally different proteins or proteins from organisms that diversified long ago. Figure 2 shows the 3D structural conservation despite low sequence similarity. structure is conserved in a monomeric hemoglobin of a trematode (PDB: 1H97) compared to a hemoglobin which is part of a large protein (3.6 million Da) from an annelid (PDB: 2GTL). In this case, the single hemoglobin from a trematode can bind and transport oxygen. However, it is structurally relevant to hemoglobins that are part of a 3.6 million-Da protein, an erythrocruorin, which serves the same purpose but has more advantages such as resistance to oxidation and other cooperative binding properties [25,26]. Both proteins are part of the globin-like superfamily [12].

In the next example, structural conservation of a plant hemoglobin (PDB: 2GNW), which may play a role in binding free molecules that cause oxidation, and a globin-coupled sensor (PDB: 2W31), which plays a role in adapting the organism in the presence of oxygen via transmitted signals to a transmembrane protein, can be seen [27,28]. This example demonstrates how a globin-like fold has been used for different kinds of responses from scavenging hazardous active molecules to sense external stimuli and cooperate with other proteins to get the appropriate response. Both proteins are part of the globin-like superfamily [12].

Nitric oxide detoxification in *M. tuberculosis* occurs with the help of a truncated hemoglobin protein (PDB: 2GLN). Its structure is similar to an extracellular giant hemoglobin from an annelid (PDB: 2ZS1) that plays a role in binding oxygen [29,30].

Certain organisms absorb light through pigments. Allophycocyanin is a pigment and its structure is part of the phycobilisome family [12]. This structure (PDB: 1KN1) is similar to a protein that plays a role in regulating the sigma (s) factor during transcription (PDB: 2BNL) and belongs to the Rsbr_N superfamily (VAST) [31,32]. This is an example of using the globin-like fold as a building block to make a larger structure like the N-domain of the rsbr to serve a different role.

The last example is from two organisms that evolved separately for many millions of years: a neuroglobin (PDB: 1OJ6) from *Homo sapiens* and a protoglobin (PDB: 2VEB) from archaea. The role of globin-like proteins in archaea is not yet fully determined. It is proposed to play a role in metabolism of the strictly anaerobic *M. acetivorans* and to be the building block of globin-coupled sensors. The structure is similar to the neuroglobin from humans which play an important role in regulating oxygen transport in neural tissues [33,34].

### Histone fold

**Figure 3:**
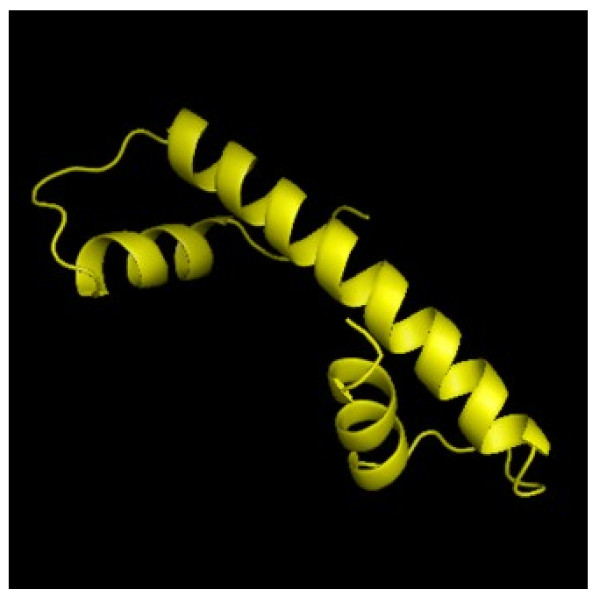
**The typical histone fold.** It consists of one central helix flanked on each side by a shorter alpha helix (PDB: 1HTA) [38].

This motif is most commonly associated with histones but can also be found in a multitude of proteins such as DNA-binding transcription initiation factors which are functionally conserved in archaea and eukaryotes [35]. Because of this functional conservation in archaea and eukaryotes, the histone fold is thought to be an ancient motif [36]. Interestingly, the pure functionality of the histone fold is not found in eubacteria [37]. As seen in Figure 3, the basic structure of the histone fold comprises a central alpha helix flanked on each side by two smaller helices.

Due to the hydrophobic nature of the histone fold, it is only stable within histone fold-to-histone fold dimers. Eukaryotic histones, for example, dimerize specifically with H2A dimerizing with H2B and H3 dimerizing with H4, thereby creating the basis of the histone octamer. Archaea histones appear to have less specificity in dimerizing to a specific partner but, through dimerization, utilize the histone fold to produce a similar histone structure [38,39].

Since the function of histones and the histone fold are shared by archaea and eukarya, it is thought to have been derived from an early thermophile which initially utilized the histone fold to maintain the integrity of DNA under thermal stress. This increased integrity would have also brought about the added benefit of genome compaction which would have required a mechanism to unwind and transcribe those genes and thus the appearance of proteins such as TATA box-binding proteins and transcription initiation factors which also utilize the histone fold and are functionally conserved in both eukaryotic and archaea organisms [35,40,41]. Since the packaging and protection of DNA is paramount along with the ability to transcribe DNA when needed, the numerous essential interactions have caused the histone fold to be conserved [42].

From a molecular point of view, the histone fold is thought to have evolved from the helix-strand-helix (HSH) motif where duplication caused two helices to merge, forming a larger central helix [36,43]. Alva et al. demonstrated how this could occur by shortening the HSH strand which led to a 3D swap and caused the dimerization of two HSH motifs. This dimerization recovered the interactions between the HSH motifs due to the strand shortening and thereby causing the histone fold [43].

**Figure 4:**
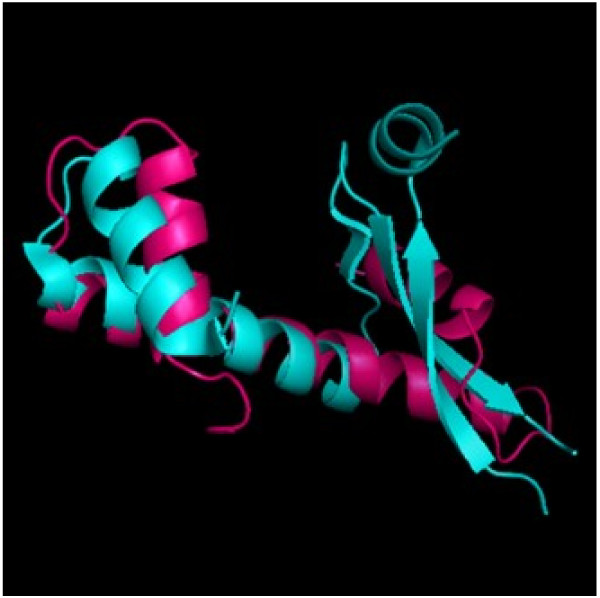
**Comparison of the histone fold (PDB: 1HTA)**[38]**to eubacteria HU protein (PDB: 1MUL)**[46]**.*** Hot pink*: histone fold, *cyan*: eubacteria HU protein. Notice the similarity in the helix-turn-helix and the size difference in the central helix.

As mentioned previously, eubacteria do not appear to contain the histone fold motif. They do, however, contain proteins which have histone-like proteins. The most ubiquitous of these proteins is the HU protein (H for histone-like and U from the U93 strain of *Escherichia coli*, in which it was identified from). HU proteins are essential in maintaining the nucleoid structure and are involved in all DNA-dependent functions [44]. Interestingly, the HSH-type motif is found in HU proteins of eubacteria which also have histone-like functionality [42,45]. Looking at the structures of HU and the histone fold (Figure 4), one can easily identify similarities in the HSH with respect to the histone fold, thereby showing how the functionality of DNA binding has been conserved through different but similar means.

**Figure 5:**
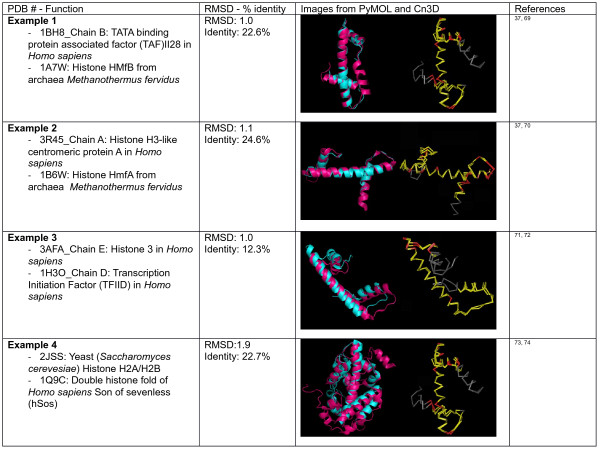
**Comparison of structure and sequence similarity of sample histone fold proteins according to PDB number.** First column: PDB number and a brief description of the protein. Second column: RMSD and amino acid sequence identity as defined by VAST. Third column: Left is the alignment of the two proteins taken by PyMOL. In the structure representation, the first protein is in *pink*, and the second, in *cyan*. Right is the alignment of the two proteins taken by Cn3D. In the sequence representation, *red* indicates the same amino acid, whereas *yellow* indicates differing amino acids. Fourth column: references.

Interestingly, some proteins have evolved a method to overcome the need of the dimerization of different proteins through a double histone fold. A double histone fold is essentially two histone folds occurring in a single peptide chain which can ‘dimerize’ with itself [47]. As seen in Figure 5, a great structural similarity between the H2A/H2B two-protein dimer has a great structural similarity to the single-protein Son of sevenless (Sos) protein [48,49]. With the double histone fold being so ‘economical’ by not needing to dimerize with another protein, it is not surprising that it was recently found in a virus where it is hypothesized to aid in the packaging and organization of DNA inside the capsid [50].

Due to the multiple interactions required of the histone fold, the selective pressures limit a large differentiation in sequence identity. For example, H3 and H4 histones are among the most highly conserved proteins in terms of sequence and length due to their specific interactions with DNA. H2A and H2B have regions which show greater variability but show great specificity to dimerizing with each other. Despite the conservation of the histone fold in the histone structure, these four core eukaryotic histones have little sequence similarity (15–20%) with one another [42]. Interestingly, even proteins such as the histone H2A/H2B and the cytoplasmic hSos [50] (Figure 5, example 4) which show strong structural similarity but do not seem to function as histones or DNA-binding factors still do not stray far from this sequence identity. This sequence similarity is seen in organisms which are obviously so evolutionary distant as archaea and eukaryotes [51,52] (Figure 5, examples 1 and 2). This may be due to the hydrophobic residue interactions required in all six helices of a histone fold dimer [39].

### Helix-turn-helix motif

HTH motif consists of an α-helix, a turn, and a second α-helix which is often called the ‘recognition’ helix as the part of the HTH motif that fits into the DNA major groove. There are several positions significant to keep the HTH structure rather than to specify contacts with the DNA, while the amino acid residues in other positions are usually varied to determine the specificity of DNA-protein interactions [53]. This motif is found in many DNA-binding domains and transcriptional factors such as homeotic proteins. This sequence, which is conserved in many organisms for related proteins, was used to discover a large number of DNA-binding proteins [54]

**Figure 6:**
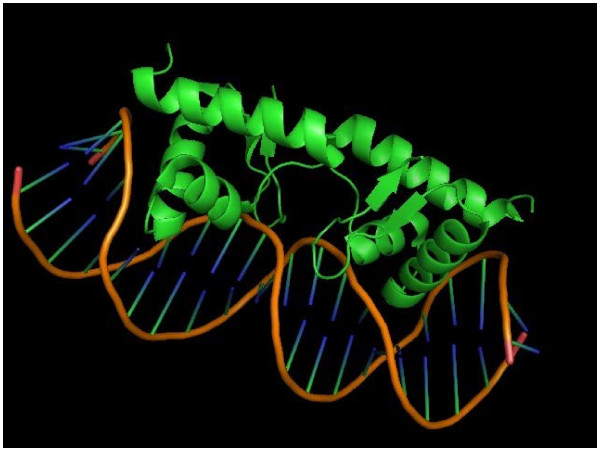
**A typical winged helix-turn-helix structure (PDB: 3JSO) **[55]**.**

Winged helix-turn-helix (wHTH, Figure 6) shares the same original ancestor as that of HTH in evolutionary history; it is also a DNA-binding domain which binds to specific DNA sequences. The wHTH is formed by a three-helix bundle (α1, α2, α3) and a three- or four-strand beta-sheet. The α2 and α3 helices are similar to those of the HTH motif except that wHTH has beta-sheet wings on the ends of HTH parts. Many repressor DNA-binding domains like LexA, arginine, Rex, ArsR, and MarR form a wHTH structure.

**Figure 7:**
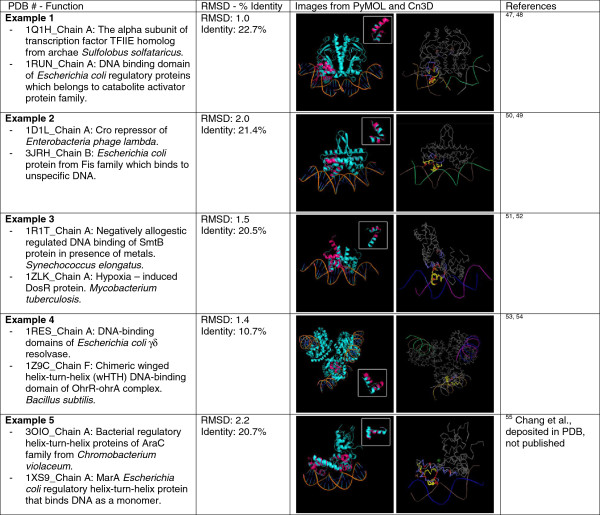
**Comparison of structure and sequence similarity of sample helix-turn-helix motif proteins according to PDB number.** First column: PDB number and a brief description of the protein. Second column: RMSD and amino acid sequence identity as defined by VAST. Third column: Left is the alignment of the two proteins taken by PyMOL. In the structure representation, the first protein is in *pink*, and the second, in *cyan*. Right is the alignment of the two proteins taken by Cn3D. In the sequence representation, *red* indicates the same amino acid, whereas *yellow* indicates differing amino acids. Fourth column: references.

Figure 7 shows five examples of HTH comparisons of different proteins. All of them show high structural similarity and low sequence identity. In addition, the examples compare HTH motifs from different organisms that do different functions.

An ancestral archaea homolog of the N-terminal of the transcription factor II E subunit a (PDB: 1Q1H) [56] folds as a wHTH. This domain has a groove which is negatively charged. Thus, it cannot bind to negatively charged DNA as in vitro experiments show. Though, it promotes interactions with other proteins. This domain has structural similarities with a catabolite gene activator protein (PDB: 1RUN) [57], a protein that is known to bind DNA. This example clearly illustrates that natural selection chose structures to have different roles than the dominant ones. Cro repressor from the λ phage (PDB: 1D1L) [58] forms a dimer by two antiparallel b-strands in order to bind to DNA. This protein has structural similarities with the bacterial Fis protein (PDB: 3JRH) [59] which binds to DNA with no sequence specificity.

Transcriptional regulators can be triggered to function by different signals from the environment. Signals that are not related with signal transduction cascades, which involve primarily phosphorylation or dephosphorylation of proteins, can involve smaller molecules like metals or oxygen. This is the case for SmtB (1R1T) [60], a cyanobacterial repressor protein that has reduced affinity for DNA in the presence of metals. The HTH motif of this repressor is structurally similar to the HTH motif of the bacterial DosR protein (PDB: 1ZLK) [61] which prolongs survival when the organism is left without oxygen.

OhrR is a bacterial protein (PDB: 1Z9C) [62] that has a HTH motif composed of eukaryotic-like wHTH, prokaryotic HTH motifs, and other helices. This protein is induced to function by oxidation of certain residues. This chimeric HTH motif is structurally similar to the HTH motif of a DNA-binding domain of a γδ resolvase in *E. coli* (PDB: 1RES) [63].

Finally, the HTH motif from a bacterial transcriptional regulator, AraC-type (PDB: 3OIO), is structurally similar to that of the transcriptional activator MarA (PDB: 1XS9) [64] which is associated with the RNA polymerase and binds to DNA as a monomer.

### Zinc finger motif

**Figure 8:**
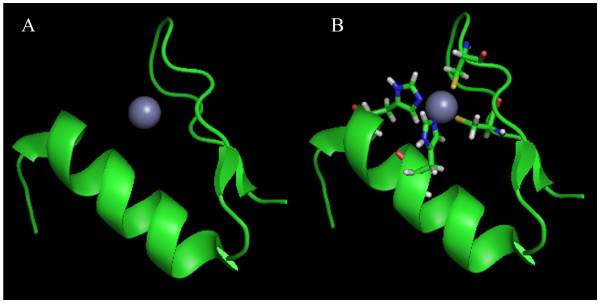
**Structure of C2H2 zinc finger of transcription factor IIIA of *Xenopus laevis *(PDB: 2HGH,**[68]**).** (**A**) Cartoon representation with zinc as a ball. (**B**) Includes the two cysteines and two histidines that interact with the zinc as sticks.

Zinc (Zn) fingers (see Figure 8) are small structural motifs whose structure is stabilized by a zinc ion, and they are the most common DNA- or RNA-binding motif in different proteins. There are different structural types of Zn fingers and are present in proteins that perform a broad array of functions such as replication and repair, transcription and translation, metabolism and signaling, cell proliferation, and apoptosis [65]. Zn fingers occupy 3% of the genes in the human genome [66]. The major part of structural stability of Zn fingers is provided by zinc coordination and by the conserved hydrophobic core that flanks the Zn binding site. There are a relatively small number of conserved residues present in Zn fingers [67].

Classical Cys2-His2 (C2H2) Zn fingers have about 30 amino acids in which 25 of the 30 amino acid residues form a loop around the central Zn ion and the 5 other amino acids form the linkers between the consecutive Zn fingers. It consists of two secondary structural units: The first one is an antiparallel beta-sheet, which contains the loop formed by the two cysteines, and the second one is an alpha helix containing the His-His. These two structural units are held together by the zinc atom. The Zn ion tetrahedrally coordinates to the conserved pairs of cysteines and histidines, and this coordination is vital for the maintenance of the overall structure of the Zn finger. The majority of the 30 amino acids are polar and basic residues which are important in nucleic acid binding. In addition to the conserved cysteines and histidines which are vital for the formation of the Zn finger fold, there are other conserved amino acids, notably Tyr, Phe, and Leu, which form a hydrophobic structural core of the folded structure [66].

**Figure 9:**
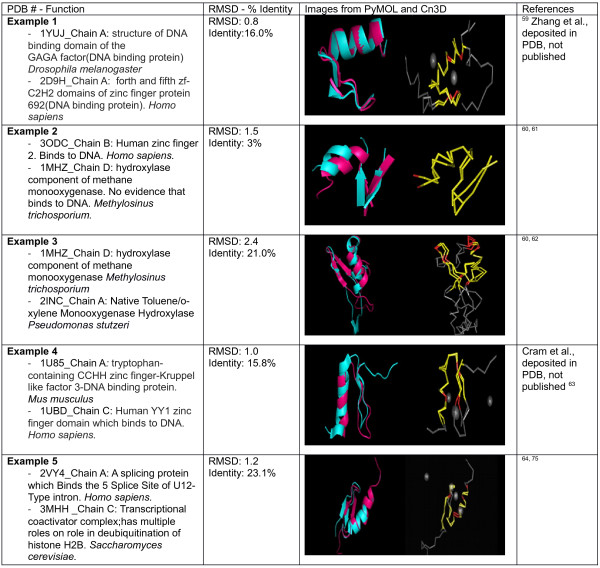
**Comparison of structure and sequence similarity of sample zinc finger motif proteins according to PDB number.** First column: PDB number and a brief description of the protein. Second column: RMSD and amino acid sequence identity as defined by VAST. Third column: Left is the alignment of the two proteins taken by PyMOL. In the structure representation, the first protein is in *pink*, and the second, in *cyan*. Right is the alignment of the two proteins taken by Cn3D. In the sequence representation, *red* indicates the same amino acid, whereas *yellow* indicates differing amino acids. Fourth column: references.

In the example shown in Figure 9, each pair of the compared Zn fingers have less sequence similarity, sometimes bind to different types of molecules, may have different functions, may belong to different species, but exhibit a great structural overlap. This supports the notion that only few small numbers of conserved residues are required for the maintenance of the overall structure of the zinc finger.

Example 1 in Figure 9 shows two DNA-binding proteins: a DNA-binding domain (DBD) from the GAGA factor (PDB: 1YUJ) [69] and one of the zinc finger domains from zinc finger protein 692 (PDB: 2D9H), which belong to *D. melanogaster* and *H. sapiens*, respectively*.* The DBD of the GAGA factor uses only one zinc finger in contrast to other zinc finger proteins which commonly use more than two in order to have a good affinity for the DNA. They show a great structural similarity despite low sequence identity.

The hydroxylase domain from methane monooxygenase (PDB: 1MHZ) [70] contains a Zn finger which does not bind to DNA. Though, it is structurally very similar (RMSD: 1.5) and their sequence is very different (3%) from the human Zn finger 2 which binds to DNA (PDB: 3ODC) [71]. This is a good example to point out that structures are built up from extensively used raw materials (domains) like the Zn finger even if they are not going to be used as the majority of the other proteins in which these domains are found.

In the third example, and as a follow up from the previous one, the two proteins are monooxygenases (PDB: 1MHZ, 2INC) [70,72] which belong to different species and have Zn finger domains whose structures overlap.

YY1 (PDB: 1UBD) [73] is a protein with four Zn fingers and is structurally similar to kruppel-like factor 3 (PDB: 1U85), which contains a Zn finger with tryptophan as shown in the fourth example.

Finally, the Zn finger in U11/U12 (PDB: 2VY4) [74], which is a RNA-binding protein, has a good structural overlap with SAGA protein (PDB: 3MHH) [75], which is a DNA-binding protein, in spite of the low sequence similarity. In addition, the role of SAGA is to deubiquitinate H2B histone, so the affinity for DNA helps to dock to the nucleosome. This example was selected because these two different proteins bind to two different types of nucleic acids, have different functions, have low sequence identity, but exhibit a good overall structural similarity.

### Concluding remarks

In this review, we have selected four protein motifs, which are present in several DNA-binding proteins and in oxygen-carrying and -transporting proteins. Using several comparisons, we show that these motifs exhibit an astonishing degree of structural conservation even though their primary sequence is not similar and even when they are involved in different functions. The examples underscore the importance of structure selection in evolution and a strategy of economy that nature is implementing. Much is to be learned when similar structures have evolved despite unrelated function. It will be interesting to determine how such similar structures have evolved and what could the possible ancestors be. Eventually, when all structures have been solved, evolution of protein structure will provide valuable information on protein function in general.

## Competing interests

The authors declare that they have no competing interests.

## Authors’ contributions

PAT conceived the idea, analyzed the data, and co-wrote the paper. KS, CEH, JC, and BS performed the search and analysis. KS co-wrote the paper. All authors read and approved the final manuscript.
